# Notes on Cases of Hydrophobia

**Published:** 1858-01

**Authors:** William Pickells

**Affiliations:** Cork.


### NOTES OF CASES OF HYDROPHOBIA;

### WHICH OCCURRED FROM TIME TO TIME IN THE SOUTH OF IRELAND, PARTICULARLY IN THE CITY AND COUNTY OF CORK; WITH INCIDENTAL REMARKS.

By WILLIAM PICKELLS, A.B., M.D., Cork.

HAVING, in former numbers of the SANITARY REVIEW AND JOURNAL OF PUBLIC HEALTH, contributed "Notes on certain Vegetable Poisons," I propose, in the present paper, to communicate notes of cases of that dire disease Hydrophobia, the result of a poison conveyed by the bite of rabid animals. My attention was first drawn to the subject by having witnessed in this city, several years since, three cases,—one from the bite of a cat, two from the bites of dogs. At the present moment, when the attention of the legislature is directed to the framing of a law, which shall more effectually restrict the circulation of poisons taken from the vegetable and mineral kingdoms, I have thought that the publication of the notes might be useful, by tending to show the necessity of more stringent legislation than that which now exists, in checking the diffusion of a poison productive of a disease which, in the opinion of the celebrated Meade, is "the greatest calamity to which mankind is liable."

The disease is commonly propagated by the dog; this animal appearing to be more susceptible of rabies than any other. In this point of view, the multitude of useless dogs that infest our highroads, and the streets and lanes of towns, is a great and alarming evil; these animals, when in a rabid state, not only biting passengers that come in their way in the public thoroughfares, but, in some instances, entering at the open doors of dwellings and biting one or more of the inmates. Nor is the evil limited to the loss of human life. Wandering in a rabid state through the country, they frequently bite, not only human beings, but cattle and other animals, valuable as property,—a consideration which should have peculiar weight in the circumstances of this island, now destined, it would seem, in the course of events to become almost wholly a cattle-feeding or pasture country. In almost all the cases of hydrophobia from the bites of dogs, which will be given in this paper, the bite was inflicted by a dog of this description.

Some medical writers have asserted that the disease, when derived from the cat, is not genuine hydrophobia, but a modified form, comparatively mild in its symptoms. Good, in his Nosology, has created two new species: 1, *rabies felina*, with little spasm; 2, *rabies canina,* with much spasm. He has omitted in his definition of feline rabies the fear of water,—the reason that induced him having been, according to the author of the article "Hydrophobia" in the *Cyclopædia of Practical Medicine,* the simple fact, that "the cases on record from cats are too few to afford a firm basis for any inference." The cases from the bites of cats, which will be given in this paper, go fully to prove that the disease, when thus communicated, is genuine, unmitigated hydrophobia. Marochetti, Christison, and Colles, are of opinion that the bite of the cat is more dangerous, as more certainly communicating the disease, than the bite of the dog. The statement of the mode in which, in some cases, the disease was contracted, owing to rashness on the part of the unfortunate sufferer, in exposing himself to the chance of being bitten, may operate as a caution to many against incurring, by like rashness, a similar fate.

In general, the disease has been communicated by strange or stray dogs or cats. In not a few instances, however, the bite has been inflicted by a house-dog or house-cat: in some instances the owner has been bitten, while unwarily fondling or playing with a favourite dog or cat; an animal fed from his table, petted, perhaps, like the lamb of Nathan in Scripture, "eating of his bread, drinking of his cup, sleeping in his bosom", becoming thus, in an unsuspecting moment, his assassin!

A few writers there have been who, setting at nought the accumulated testimony of so many ages, and of so many various nations, have denied the existence of hydrophobia as a real specific disease, regarding it as, in every instance, "the result of fear,"—a thing of the "imagination"—a metaphysical abstraction, existing, according to one, "only in the brains of a few speculative writers;" according to another, "a fable for the historian, and a metaphor for the poet." A fair appreciation of the facts of several of the cases, which will be brought forward in this paper, might, if other evidence were wanting, be alone sufficient to show the fallacy of so dangerous and Quixotic a notion.

I was particular in noting instances from the bites of cats; the cases on record from this cause being, it appears, so few.

The sufferer in the case from the bite of a cat which I witnessed in this city, and with the details of which I shall commence, was a fine robust boy, of the name of Lynch, fifteen years of age, son to a poor inhabitant of the North Parish. Seeing, at an early hour in the morning, in the month of April

1830, a cat run across one of the quays of this city, and enter a hole in a waste house, he pursued in order to catch it for the use of his family; but upon putting his hand into the hole for the purpose, he was instantly bitten in the back of the fingers. The animal continued to adhere to the fingers, though the boy withdrew his hand, until repeatedly dashed against the ground. After this, the cat disappeared among the ruins of the waste house, and was no more heard of. The fingers continued to bleed freely during the rest of the day; but, as they healed in the course of some days, without any application, the occurrence passed unheeded. In about six weeks afterwards, on a Monday, the bitten hand, after fatigue in playing ball, became swollen so that he could not close nor lift it; he had also pain extending along the corresponding arm to the throat,—"along the veins of the arm," was the expression used by himself and his family, to mark the direction of the pain. There was no tumour under the axilla. On Wednesday morning following, the symptom of dread of liquids developed itself. There was no application, however, for medical assistance until Saturday evening following,—that is, until the evening before the day of his death, neither himself nor his family suspecting for an instant the real nature of his complaint. I saw him on that evening, at the house of the late Dr. John Barry, of this city, to whom he had made applition as for a common sore throat, accompanied with inability of swallowing. Upon examination, however, of the uvula and fauces, no inflammation was observable. This circumstance, coupled with the boy's statement of inability of swallowing liquids, at once roused suspicion, and led to the eliciting of the facts which I have here stated. He did not lay the slightest stress on the circumstance of the bite, which was compared by himself and his family to scratches of a pin, nor would he have mentioned it had he not been asked, having almost forgotten the occurrence. Scratches were still evident on his fingers. He had walked from his father's house, a distance of nearly a mile, to Dr. Barry's, and had been sitting half an hour there when I saw him; he was very communicative, I might say voluble, in conversation; professing his readiness to submit to any mode of treatment recommended. I had never seen a case of the disease before, and my curiosity was the more awakened, as I was aware, at the time, that some medical writers had denied the communicability of true hydrophobia from the bite of a cat. On being questioned, he said that since Monday morning he had not swallowed a drop of drink, nor tasted a morsel of food; adding, that he would give the world to be able to take a drink of water, but that he "had a dread of it, that it stopped in his throat." What struck me most, was the excessive nervous agitation of his whole manner, indicative of the utmost misery and horror. Next to this, what appeared most remarkable was the very peculiar expression of the eye, denoting something indescribably scared and haggard. The pencil of an artist might have portrayed it, but words could not have described it. In tone of voice and conversation, however, he appeared bold and undaunted, not entertaining, seemingly, the slightest consciousness of the nearness of death. He complained greatly of oppression of the præcordia, or, as he expressed it, "a drag about the heart"—the heart, as I found, on pressing my hand, palpitating violently (thumping, as it were) against the pleura. His shoulders were much elevated in the effort of respiration. This symptom, however, he attributed to a play-fellow having, some weeks before, raised him from the ground, and held his hand to his mouth until he almost suffocated him. There was no headache, no quickness or other irregularity of pulse. The tongue was slightly furred; the bowels were costive. There was no derangement of the urinary secretion, which was passed freely without pain. Convulsive paroxysms followed, upon his being presented, first with a glass of milk and afterwards with a glass of wine. I can never forget the peculiar oblique look, and suspicious scowl, with which he eyed the liquid as it was brought near, until, at the moment when it came directly opposite to him, he started, as it were, with horror, averting his head, crying, "Oh! oh!" his throat swelling at the same time with spasm. There was no spitting as yet; none of the sublingual glands, described by Marochetti and others, was observable upon examination. The sensibility to cold air, or a current of air, was such that "the draught of a person passing by" affected him. He was quite unaffected, however, by the light of a large window, to which he continued to sit opposite for nearly an hour. While sitting at Dr. Barry's, a member of the doctor's family remarked that the sight of the big drops, which sometimes trickled from his perspiration, brought on the spasms. He was conveyed home in a car, covered, at his own request, with a woman's cloak, though the evening was mild. I saw him on the following morning, in company with Dr. Barry, at his father's house. He had passed a sleepless night. An enema of four ounces of turpentine, which we had ordered, brought off two stools. A warm bath, which we had also ordered, afforded him, he said, some relief. No convulsions took place during immersion in the bath, nor during the administration of the enema. He had sucked, before our visit, about half pint of water through a wheaten straw, and had also eaten or sucked part of the pulp of an orange. His pulse was now evidently become weak. There was some spitting, and occasional vomiting of white frothy mucus. He continued to complain greatly of want of breath and air. The cheeks were flushed, the face and neck drenched in perspiration. His hair, it was observed, stood erect (*horripilatio*). Upon visiting him two hours afterwards, I found his position in bed had been reversed, at his own request, to avoid, as he said, though the day was very mild, the air from an opposite hole in the wall, which served for a window. He had raved for a moment or so in the interval since the former visit, having fancied that an apron, which hung in the room, was a black cat, and, under this delusion, causing it to be removed. The spitting had now become constant, and the vomiting of frothy mucus very frequent. He consented, to oblige me as he said, to repeat the attempt of sucking in water through a straw, trying for the purpose different straws, until he hit on one of such length as, with the extremity dipped in the vessel, not to require him, while sucking by it, to bend the neck. During the trial of these different straws he caused the vessel to be placed on one side, not directly opposite to him, so as to avoid the sight of the liquid,—refusing, however, to allow his eyes to be bandaged, lest it might "confine his breath." In this way I saw him suck in laterally, his eyes steadily averted from sight of the liquid, about a noggin of water, stopping short at intervals to take breath. His face and neck continued to stream with perspiration. Vinegar has been extolled by some as a specific in the disease. He attempted to suck in a little weak vinegar; but, after swallowing a few drops, was immediately forced to desist, crying out that "it burned him within." On the evening before, while at Dr. Barry's, he could bear to look, though not without repugnance, upon a mirror which was presented to him; but upon a fragment of one being now presented, he turned from it with aversion. He died tranquilly in two hours afterwards, without convulsions or hiccup, manifesting no inclination to bite or spit at any of those present. Some minutes before death, he called for the family prayer-book, and read, or seemed to read, a portion of the contents. Though, during the last hours of his existence, labouring under a state bordering on delirium, with the exception of the delusion of fancying that an apron which hung in the room was a black cat, he shewed no symptom of actual delirium throughout. He lived six days from the commencement of constitutional symptoms, and somewhat more than four from the development of the symptom of dread of liquids. A *post mortem* examination was not allowed. Upon my calling at half-past 12 on the following day, putrefaction, I learned, had already commenced, particularly in the bitten hand.*

With the case of poor Lynch may be coupled one in which the disease was contracted under like circumstances of rashness. It occurred several years since near Clonakilty, in this county, as communicated to me by Dr. Callinan, of this city, who witnessed it, and was at the time a resident of the place. A peasant, seeing a strange cat on the road side, at some distance from his house, as it is always an object with the lower orders to make prize of a stray cat, thought to catch it; but in his making the attempt, it bit him in the hand, adhering so firmly, that he found considerable difficulty in extricating the hand. The cat was killed rabid. He was a foolhardy sort of fellow, and thought no more of the accident; people in general, as Dr. Callinan justly remarked to me, being little aware that the bite of a cat may occasion the disease. In about six weeks afterwards, characteristic symptoms were developed, preceded by the return of inflammation and swelling in the bitten part. The wound did not heal; a scab remained on it. Around the bitten spot was a dark areola. He died horribly delirious. The sensibility to atmospheric exposure was felt as a severe aggravation of his sufferings; a current of air which blew on his bed-place from a hole in the opposite wall of the cabin, which served for a window, agonising him exceedingly. There was much enlargement (tumefaction) of the region of the stomach, as from flatulence.

About twenty years before the occurrence of the case of Lynch, occurred the case of the servant of a principal merchant of this city, Mr. Goold; the man was bitten by a strange cat, which he struck with a napkin while returning after carrying in the dishes to table. The case excited at the time great interest, and was seen by a number of physicians. Dr. Barry, who was one of the attending physicians, informed me that the nervous sensibility was such, that when he passed between the patient and the light of the window, the man would start up out of bed involuntarily as if to seize him, but would lie down again immediately and apologise. The noise of water poured from one vessel to another brought on the spasms. There

---

* Happening, shortly after the occurrence of the case of Lynch, to attend professionally in the family of a principal baker of the city, on mentioning to him the facts of the case, he told me he never made use of a cat, as he found an *owl* more efficient in keeping the bakehouse clear of mice. An owl is said to be worth six cats in keeping a barn clear of mice.

was great spitting, and, towards the end, incessant plucking of the bed-clothes as in the last stage of malignant typhus.*

Some years afterwards occurred the case of a Mr. Cronin, who, travelling to town from Mallow, in this county, went into a waste house on the road side, when, just as he entered, a cat sprang across and bit him in the foot. In about six or seven weeks afterwards he was seized with symptoms of hydrophobia, and died, as I learned from his medical attendant, in forty-eight hours, in convulsions. The sight of a mirror alarmed him.

In each of the cases now given, the bite was inflicted by a strange or stray cat. In each of five other cases, all those of females, and which occurred in this city, the bite was inflicted by the house cat.

In the instance of Miss Tisdall, a child eight or ten years of age, which occurred many years since, the animal, I was informed by a near relative, had, the evening before, devoured its young. It had found access, somehow in the morning, to the child's bed-room before she rose, and had hidden itself under the bed. Upon her stepping out of bed, the animal sprang at her from under the bed and bit her in the thigh. She lived about a week from the commencement of the sickness. After inflicting the bite, the cat allowed itself to be brought down stairs, and without attempting to bite any one else, was hanged.

Some years before or after, occurred the case of Miss Gray, who was bitten while turning a cat out of her room previously to retiring to rest.

In another instance, Miss Bennett, an elderly lady, while playing with a favourite cat, was bitten by it in the throat. Hydrophobia developed itself, according to my informant, in three weeks afterwards. It is certain, that this lady was suffocated at her residence, in this city, between beds.

In another instance, which was seen by Dr. Baldwin (sometime representative in Parliament for this city), a Mrs. Crowley, after having the cat in her lap, put it down. The animal wanted to get up again. She struck it. The cat flew at her, scratched and tore her face, and bit her in the leg, adhering with such tenacity to the part, that the servant who came to her assistance, could detach it only by cutting its

* The quickness of ear of hydrophobous patients, in the perception of sound associated with the idea of water, was curiously illustrated in the case of a boy, as mentioned to me by the attending physician, which occurred several years since near this city. Not only the noise of empty tin vessels, though in another room, brought on the spasms, but even the noise of a horse drinking at a stream, which crossed the road at some distance, though no one else in the room could perceive the noise, and though he sat upright in bed!

throat. In six or eight weeks symptoms of uneasiness came on. She was so delirious that it was necessary to tie her to the bed. She also was, I learned, suffocated between beds, a practice formerly permitted from a mistaken motive of humanity, to put an end to the patient's sufferings; ("the patient," as observed by Boerhaave, "dying soon enough without accelerating his fate"); but which has been now expressly prohibited by law in most countries of Europe.

About the same time, a case occurred in the Munster Hotel of this city, as mentioned to me by the attending physician, of a chambermaid, who died of hydrophobia from the bite of the house cat.

Since the case of Lynch, in 1830, no case of hydrophobia from the bite of a cat has occurred in this city. Several have occurred within the period from the bites of dogs, and one from the bite of a fox.

In this county, in the town of Macroom, occurred in February last, 1857, the case of a Mrs. Shea, the wife of a baker in the town, mother of a large young family, "who," according to the report of the attending physician, "was employed in her shop on or about the previous September, when a young cat ran in from the house of a neighbour. She endeavoured to drive it out, but it took refuge behind the counter, and, fearing that it might jump through the window, she pursued and caught it, and when in the act of putting it out of the door, it turned on her and inflicted a severe bite on the little finger of the right hand. She at the time expressed some uneasiness at the occurrence, but soon forgot it, and continued to enjoy her usual good health until the 4th of February, when she complained of pain running up her right arm. She died on the morning of the 8th. Chloroform administered, so far from relieving, increased the convulsive suffocation to such a degree, that she declared it would cause her instant death if not removed. She would not permit the bottle to remain in the room."

In the workhouse of Mallow, in this county, about a year and a half since, occurred the case of a child who, it would appear, was not bitten, but only scratched by a rabid cat. The scratch of the claw of a rabid cat has, it is stated, communicated hydrophobia to the human subject. A coroner's inquest held on the child returned a verdict that she died of hydrophobia.*

In the autumn of 1843, a case occurred in Tralee, county

* The spur of the ornithorynchus paradoxicus is, according to Blumenbach, the only instance of any part of an animal in health being capable of conveying hydrophobia.

of Kerry, of the death of a grown boy from the bite of a cat. The cat had strayed from the house; the boy pursued it and seized it, when it bit him.

Braugmarten, a German physician, who has written on hydrophobia, says, "Before the dread of water sets in, the cure is not only practicable, but not unfrequent." He seems to have echoed the words of Avicenna, who says in a remarkable passage, speaking of hydrophobia:—"Cura propinqua est ante terrorem aquæ." If stress can be laid on a passage, the terms of which are so vague and ambiguous, it must mean the interval between the commencement of constitutional symptoms, and that of the dread of liquids; for, previously to the appearance of constitutional symptoms, there is nothing to indicate that the disease will take place. An early indication of the virus having become active, or of the constitutional action having commenced, is not infrequently afforded by the reappearance of inflammation or pain, as in the cases of Lynch and of the boy near Clonakilty; or by numbness or prickling sensation (as in a case which will be subsequently given), in the bitten part; analogous to the effects of other diseases which can be communicated by inoculation, it appearing to be a law of such to excite inflammation, or recrudescence, at the place of insertion, shortly before they shew themselves in the constitution. I find it stated too, by some eminent recent authorities, that if any good can be done, it is in this stage; in the second stage, that is, when the dread of liquids has set in, all means hitherto used having proved unavailing. In Lynch's case, the interval lasted two days, that is from Monday till Wednesday morning; but it sometimes exceeds this period. The misfortune, however, is, that no application is made for medical assistance until the disorder has been fully developed, or has passed this stage; it being mistaken, as in the cases of Lynch and of Mr. Cronin, for a common sore throat; or, as in other instances, for a cold or feverish attack, or for rheumatism, or hysterics, or some other affection, masking the real disease. The pain and swelling of the bitten hand were attributed by Lynch and his family to over fatigue in ball play. It may be a question, whether but for the excitement of the ball playing, the virus would have been called into action at all; an observation which suggests the necessity of caution to those who have the misfortune of being bitten by a rabid animal. A medical writer states, that, "in all the cases he had met with, there had been shortly before the setting in of the symptoms, exposure to some considerable excitement."

John Hunter, in an able paper on the disease, in remarking on the little diminution of muscular strength, in the operation of a poison so destructive to human life, gives two cases, which though they ultimately proved fatal, "were remarkably relieved by running." The relief thus obtained could not, however, as may be inferred, have been owing to the perspiration excited; a supposition, to which what we are told of the effects of dancing in curing the disease occasioned by the bite of the tarantula, might seem to lend a degree of plausibility, as numerous cases have been attended, as in the case of Lynch, with copious and long-continued perspiration without relief having been obtained.*

Of the two cases of hydrophobia seen by me, in each of which the bite was inflicted by a dog, one was that of a female, the other that of a male. The female, a young woman of about eighteen, had been bitten four months before by a *cur dog*, belonging to an inmate of same house, the house being tenanted by poor room-keepers. The case was seen also by Sir James Pitcairn, late Deputy Inspector-General of Hospitals for Ireland, who resided at the time in this city. The case occurred in 1832. I saw her on a Thursday, about one o'clock, p.m. Symptoms commenced with pain and sense of suffocation in throat, but she did not take to her bed until Tuesday night. She had been hot and feverish for a couple of days before. She could not swallow any drink—(she had eaten, however, two or three strawberries). The moment a vessel touched her lips, spasms came on. When spasms came on, there was increased action of the heart, with occasional, as ascertained by feeling the artery, in-

* M. Buisson, in a small tract addressed to the Paris Academy of Sciences, in 1823, using as an argument from analogy the story of the mode of cure of the bite of the tarantula, recommends as the mode of treatment in hydrophobia, "that the patient should take a certain number of vapour-baths, and should induce every night a violent perspiration, by wrapping himself in flannels, and covering himself with a feather bed." Those who seek to cure hydrophobia by forcing sweats, whether by violent exercise, or by the vapour-bath, or by the hydropathy of the present day, seem not to be at all aware that sweating as a symptom of hydrophobia, had been noticed so far back as the time of Cælius Aurelianus. Lynch's fancying an apron that hung in the room was a black cat, and under this delusion causing it to be removed, might seem to lend a degree of plausibility to the above supposed analogy; persons bitten by the tarantula, according to Baglivi, having a great aversion to black. In one of three cases seen by Dr. Meade, as reported by him in vol. 5 of "Phil. Transactions", abridged, the patient, a boy, "had a strange aversion to anything white, saying that, if all the women in the room who had white aprons would go out, he would be well presently." Meade attributes this to hydrophobous patients so ill bearing the light, that the sight of anything white is intolerable. In Lynch's case, however, there was no intolerance of light. Truth is, that though all, with very few exceptions, agree in the main symptoms of dread of liquids, and dread of air (hydrophobia and aerophobia), the difference in minor points, in different subjects, is such, that in this respect hydrophobia, as has been said of typhus fever, may be said to be a Protean malady.

termissions of pulse. When the spasms went off, the pulse became regular again, with a rate of about 130. Dread of cold air was very distressing. She cried out that some person in the room had a cold breath. There was intolerance of light. When a looking-glass was presented, she could bear to look at it, but said her eyes, which were naturally full and prominent looked "like moons." Previous to the present attack, she was described as being remarkably beautiful, and of rather a full habit; her features were now sunken and ghastly. Towards the end there was great discharge of saliva. There was no actual delirium, but, as Dr. Pitcairn expressed it, "she seemed every moment ready to fall over into the sea of delirium." She cried out "to be tied." She died on the same night at ten o'clock; that is, in five days from the commencement of constitutional symptoms. She was bitten in two places, in the fleshy part of the right leg. She complained of pain extending from the bitten part down her leg; the ankle of the bitten extremity was blue. Turpentine enemata were the curative means tried. The dog, after biting another inmate of the same house, was hanged.

Thomas Keeffe, aged 17, of robust frame, living in Hardwicke Street, was seen by me on Tuseday, August 12th, 1834. His father was holding him when I went into the dark narrow back ground-floor room, in which he lay. He cried out to me, "they were restraining him," calling them murderers. He thought the bite of "that dog" did not signify. He had been bitten about three weeks before by a strange cur-dog, which came into the room from the street, and to the tail of which he attempted to tie a canister; it being a mischievous trick of boys to tie a canister to a dog's tail, and then to send him running a-muck with it through the streets. He was bitten in the thumb of the left hand in three places; the cicatrices were small, though it had been found necessary to detach the dog with a shovel. The wound did not fester, but healed readily, and the thing passed unheeded by himself and family. On the previous Sunday morning, after a sleepless night, feeling himself thirsty, he got out of bed to take a drink from a water-jug, but, on attempting it, "shuddered, and could not touch it." He ate his dinner apparently well on Saturday; the night before, however (Friday), he had also a sleepless night. He walked on Monday morning to Dr. Harvey's house in a neighbouring street. There was great anxiety, Dr. Harvey told me, about him then, but he did not suspect what was the matter. He was struck, however, by his desiring the woman who came with him to cover his mouth with her cloak. Shortly after his return from Dr. Harvey's, he was visited by a clergyman, who, surprised at something that occurred, while administering the rites of the church, was led to inquire of him whether he had been bitten by a dog. It was thus that the real nature of the disease was discovered, and communicated by the clergyman to his family.

It was about three o'clock, p.m., when I saw him. He had taken some pills, ordered for him by Dr. Harvey, but had not swallowed any liquid since the previous Saturday. He made frequent attempts by "coaxing it sideways to his lips," and with various gesticulations, somewhat in the manner of those affected with St. Vitus's dance, but failed. In the progress of his sickness, the morbid sensibility of surface was such, that even the cold breath of a person who approached him agonised him. As in Lynch's case, urine was passed freely and repeatedly, without pain, or without, as sometimes happened, bringing on the spasms. The discharge of saliva and frothy mucus was very profuse, the floor being all begrimed with it. There was no actual delirium, but the excitement was so great as to border on it. He at one time cried out "to tie him;" was quite aware of the nature of his disease, frequently exclaiming, "I am innocent of that dog," and seemed apprehensive of being smothered, exclaiming, "they are going to murder me," "I am dying," "Let me die." Words of his at intervals to those about him, though wild and incoherent, were painfully affecting. "Father, kiss me, though you are my murderer. Don't kiss me, for fear I may bite you, and you may go mad too: kiss my foot." He called on us individually by name to pray for him, and say, "Lord have mercy on him." His last words were (to his sister), "Hannah, good bye." I forbade his family to kiss him. "Father, they will be yet throwing this in your face"—his having died of the bite of a mad dog. He died tranquilly at half-past six the same evening, on the fifth day from the commencement of constitutional action, and on the fourth from the development of the symptom of dread of liquids. Upon calling on the following day, putrefaction, I found, had commenced, the thumb and palm of the hand being livid, and a black line or streak extending from the thumb up the arm to the acromion. Might this have been an indurated or enlarged lymphatic? Monro (*On Morbid Anatomy of Gullet, Stomach, and Intestines*) says, "Though it is probable the poison of hydrophobia is absorbed, yet the lymphatic vessels, and lymphatic glands have not been observed to be indurated or swelled." The morbid action of the poison is still "a vexed question;" whether it is carried into the circulation by the lymphatics, so as to effect a change, leavening, as it were, the whole mass of blood; or whether it causes some peculiar effect upon the nerves of the injured part, and through them on the brain and nervous centres.

In both the above cases, there was a remarkable preference of the prone position in bed, apparently with some relief to the respiratory organs, dyspnœa having been in both a prominent and very distressing symptom. In two or three instances in which typhus fever was complicated with asthma, I remarked a like preference of the prone position, as if affording some relief to the respiratory organs. In Keeffe's case, while in this position, there was much jactitation of the lower extremities.

In none of the three cases which I saw, was it found necessary to resort to any extreme measure of coercion. In Keeffe's case I had been strongly urged by his family to allow the application of the strait-waistcoat, but refused, conceiving that in a case so utterly hopeless, in which there was no disposition to injure others, it would have been a measure of unnecessary rigour. Without going the length of saying with one of the medical gentlemen, examined before a committee of the House of Commons, in 1830, on the subject of canine madness, "It is a vulgar error to suppose hydrophobous patients are mad; they are not mad," I believe the madness, or furious delirium, attributed to them has been often the exasperation produced by needlessly harsh treatment.

In the great hospital of Paris, it was formerly the rule to tie every hydrophobous patient indiscriminately to the bed. In Prussia, by an edict of the present king, some years since, among other directions for the treatment of hydrophobia, it was ordained, that every hydrophobous patient should be confined in a strait-waistcoat.*

The following case which occurred in July, 1835, at a place called Feenagh, county of Limerick, as reported by the dispensary physician, the late Dr. Graham, evinces the prodigious augmentation of muscular strength which, when the symptoms *are* maniacal, sometimes takes place in hydrophobia. The sufferer was a lad about twenty years of age, named Garrett Nagle. He "was working on the repairs of a road about three weeks before, and was bitten in the hand by a *small dog*. From that time until the afternoon of the day preceding the night on which he died, no effects were apparent; but now he complained of drowsiness and headache. He left off work and came home,

* In the Prussian dominions, in the ten years ending in 1820, 1666 persons, according to Marochetti, died of hydrophobia.

when his mother prepared some mulled porter for him. At sight of the liquid he shewed some uneasiness, and when it was offered to him to drink, he became delirious, and shortly afterwards raging mad. He was secured and tied to a bed by two policemen (his relatives and friends, as well as all the neighbours, having run away from him), but he broke both the bindings and the bedstead to pieces, and he was, at his own request, during one of the lucid intervals from the violent paroxysms of spasm, handcuffed by the police to prevent his injuring himself and those who were about him. He continued in the most excruciating torments for about sixteen hours, when death released him from his sufferings."

The mention of the police being employed in the above case, brings to mind a case which occurred, several years since, in a barrack, as communicated to me by a friend of mine, an army surgeon, who was quartered at the time with his regiment in the barrack. The dog, a sporting one, of the spaniel breed, had entered the barrack by night and bitten one of the men, who afterwards died of hydrophobia. It had been turned out of an upper room which it had entered, in which were a number of the men. The unfortunate man who perished, having had occasion to go down stairs, was met by the animal on his return, when it flew at him, and bit him in various places before it could be bayoneted. Upon dissection of the dog by my informant, he found in the stomach, besides the usual crude mass of indigestible matter (feathers, straw, &c., which the animal had gobbled up) half of a fowl (a hen), with the feathers on. The stomach was quite dry and contracted, as if the secretion of the gastric juice had been completely at an end. The membrane, he thought, somewhat thinner than natural. There was a red patch or two about the cardiac orifice. On the pia mater was a slight blush of inflammation, but no effusion. There was no inflammation of the trachea or œsophagus. An assistant in the dissection, happening to have a scratch on one of his fingers, being of a nervous temperament, fancied he had inoculated himself with the virus, and was seized with hysterical paroxysms, resembling those of hydrophobia, which lasted two or three days.

Since the case of Keeffe, in 1834, I am aware of five cases from the bites of dogs having occurred in this city, or its immediate vicinity; all those of children so young—from five years of age to eight—as to preclude the idea that the disease had been brought on by fear or mental emotion, arising from knowledge of or sensibility to the peculiar danger incurred from the bite.

In 1837, occurred the case "of a boy, five years old, who died in the South Infirmary of this city, after fifty hours suffering. He had been bitten by a dog five months before," the symptoms having been more tardy in supervening than is usual in young subjects.

After an interval of nearly ten years, there occurred in March, 1847, the case of a girl, eight years old, who was bitten in her own dwelling by a cur dog which leaped from under a table and bit her severely in the face, breaking three teeth of the upper jaw. The symptoms began to manifest themselves four weeks after the occurrence. She lived four days. There was little or no mental aberration. Though disinclination to liquids was marked, there was no actual inability of swallowing them. The most striking symptom was dyspnœa. The wound had been attended to by a surgeon and soon healed, there having been no unhealthy discharge.

In each of three other cases which occurred subsequently, the child was said to have been bitten while at or near the door of its dwelling, by "a small dog"; in one instance a poodle, which ran by, snapping in its way. The last case was in the spring of 1854. Thomas Barry, a child five years old, son to a farmer living near town, was playing with some other children in a haggard, when a large dog, in running by, snapped at and bit him in the nose. The wound was little more than a scratch, and healing readily, gave no alarm; it happening, indeed, not infrequently that when the injury has been very slight, the bite is almost as little regarded as a flea-bite. He continued to go daily, as usual, to a neighbouring school. In about six weeks after, while at school, he was seized with a fit of crying, and was otherwise so troublesome, that the mistress directed an elder child, a sister who went also to the school, to take him home. In doing so, she thought to avail herself of a short way which led across a small stream of water or ford; but on coming to it, at sight of the water, the child recoiled, screaming, and could not be made to pass! It was found necessary to carry him home by another way, so as to avoid the sight of the water, on a man's back. In the course of his sickness, though "dying," as one of the family expressed it to me, "for a drink of water", when brought to him, "he could not look at it, nor touch it." He lived about thirty hours from commencement of the symptoms. He died in great agony. He was quite delirious. He foamed much at the mouth. Under the delusion of an old prejudice, which unfortunately has not yet become quite obsolete even among the better classes, he was said to have barked like a dog. The dog was shot A dog and calf, which were also bitten by it, died rabid.

In February 1833, occurred a case from the bite of a fox; the bite having been inflicted four months before. The sufferer, a man named Driscol, had risen at an early hour in the morning in order to be in time for his work at Lota, three miles from his place of abode. A little dog he had with him pursued an animal into a sewer on the highroad. Supposing it to be another dog, the man hallooed. The fox bolted, and after running a little way, pursued both by dog and man, turned back and flew at the man, biting him in the tip or lobe of the left ear. He at first repelled the attack; but in the second attempt was bitten. He paid little attention to the accident himself, until the ear was seen bleeding by a young woman going the road. The fox, after biting him, ran back into the sewer, and was coming out again to attack him, but went back. The man lived from Monday till Thursday. On Monday, when my informant, the late Dr. MacNamara, first saw him, he would hold, while conversing with him, a vessel of water a considerable time in his hand, but when he attempted to drink the paroxysm came on. At another time, the bare mention of water brought on the spasms. The morbid sensibility of surface to currents of air, or the impulse of any thing blowing on him, was present to a great degree. He could not bear the sight of a mirror, nor a piece of sheet tin, when presented. He lay quiet. It was not found necessary to restrain him. He retained consciousness to the last. According to his dying request, the sewer was examined, and the skeleton of the fox was found there. It was a pet fox, supposed to have strayed from a neighbouring gentleman's demesne.* Many years since a case occurred in this county of a huntsman, who got hydrophobia from the bite of a fox, in endeavouring to save the fox from the hounds.

Arising almost always from dogs, the spread of hydrophobia in this part of Ireland has been commonly traced to the *cur-dog.* The multitude of dogs of this sort on the high-roads, and in the streets and lanes of towns, has been long complained of—to quote words applied to it—as "a crying and enormous evil." In many of the lanes of this city, scarcely is there a room-keeper, however poor, who does not keep one or more dogs of this sort, to the danger or terror of the passenger in passing through them. The dispensary physician,

* The young fox, by the bite of which, while patting it, the Duke of Richmond lost his life in Canada from hydrophobia, was a pet fox.

in visiting the sick poor at their homes in these lanes, incurs, as he has often complained, considerable risk, these yelping inmates being apt to fly at a stranger. When the owner is expostulated with, and asked how it happens that he who can hardly feed himself, keeps so many dogs, the answer is,—"They cost nothing, sir; they forage for themselves:" that is, they prey on the garbage thrown out from houses by night into the streets, and this failing, take to plunder, snatching a mouthful, when opportunity offers, from some kitchen, or cookshop, or huxter's or butcher's stall.*

In the two cases of hydrophobia from the bites of dogs, which I saw in this city, the bite, as mentioned above, was, in each instance, inflicted by a *cur-dog.* At dates prior to the occurrence of these cases, occurred the case of a poor sweep, who got hydrophobia from the bite of a cur-dog in separating him from another dog, with which he was fighting in the street;† and that of a child, who was overturned and bitten over the eye by a cur-dog, in making his escape from another with which he had been fighting in the street. The disease came on in three weeks after.

The writer of a letter signed a "Friend to Humanity," addressed to the editor of the morning paper of this city, in March, 1848, in communicating the fact of a cur-dog, apparently in a rabid state, having a day or two before run through the most populous streets of the town of Bandon, in this county, and bitten no fewer than ten persons, before he was destroyed by the police, added, as showing the necessity of more stringent legislation, that besides cases of hydrophobia occasionally noticed in print, many others had occurred in the western parts of the county, without having received publicity.

In Limerick city, much about the same time, "a large black dog, in a rabid state," according to a Limerick newspaper, "entered the house of Mr. ——, Thomond Gate, and bit the servant boy, who was in the kitchen, in the thumb and shoulder. The furious animal then ran down Thomond Gate, snapping at every one in his way, and effected his escape without being destroyed.". Two of the individuals bitten died some time after of hydrophobia. In Limerick city, some years

---

* "He that has not bread to spare, must not keep a dog", is a Spanish proverb: "A quién no le sóbra el pan no crie can."

† In Durham, in England, some years since, a Mr. —— died of hydrophobia, caused by the bite of a dog, which he endeavoured to separate from another, with which it had been fighting under a table. So true it is that, "He who parts a fray, gets the worst of it."

since, as stated in the newspapers at the time, "One of the city police having discovered a pig, quite in a rabid state, in one of the streets, shot it, and had the body thrown into the Shannon. In the course of the evening some wretches attempted to take it out of the river, in order to expose it for sale. The police having been informed of this movement, had the body taken up and burnt." There can be little doubt that the flesh of animals which have died rabid is often sold in the market. It is a moot question, whether the flesh of animals, which have died rabid, is not, when eaten, capable of communicating hydrophobia to the human subject.*

In country districts, the multiplication of cur-dogs appears to have been a still greater nuisance than in towns. The traveller could pass scarcely a farm-house or cottage, however poor, without being annoyed and worried by one of these inmates rushing out. They were snappish, and to be found at the heels of every horse; by alarming with their noise, and sometimes seizing the animal by the heels, causing him, in not a few instances, to run away with his rider. Parties of idle fellows might be seen, particularly on Sundays, accompanied by troops of these dogs, and of terriers, roving through the country in search of game, or for the purpose of baiting cats, though "a baited cat has sometimes turned a man into a mouse, becoming fierce as a lion." In one of the volumes of Martin Doyle's *Quarterly Journal of Agriculture*, published some years since in Wexford, is "A Chapter," so entitled, "on Cur-dogs." After a rough calculation or census of the comparative numbers of other dogs, the writer observes: "We have 10,000 dogs left, which I shall beg leave to call useless, troublesome, and food-consuming curs. On my entering the cabin of a poor widow and her family, living on a meal a day, a dog lay cowering near the hearth. Of course he flew at me on my entrance. Why, said I, do you keep this brute as sentry, when you have not as much food as would give supper to a mouse? The widow assured me, in reply, that the cur was not an expensive inmate, that 'he was a good warrant to shift for himself, and that she never gave him a bit to eat;' in other words, that the poor brute was left to prowl about the country in search of a mouthful of carrion, which he may

---

* Nothwithstanding our civic authorities' fulminations against unmuzzled or unlogged dogs, and our canicidal campaigns in the dog-days, as if hydrophobia and canine madness were not of all seasons, to how many cities and towns may not, at the present day, the line of Horace in reference to the streets of ancient Rome apply:—

"Hâc rabiosa fugit canis, hâc lutulenta ruit sus!"

get in two months, he being otherwise led into the temptation of taking a lamb or sheep from a neighbouring field, or of preying upon a farmer's fowl yard. Many a horse," the writer adds, "has, in this country, started and kicked, and run away with his rider, and flung him to the ground and killed him, in consequence of the sudden rushing out of a cur-dog at his heels; and many a man has been deprived of life in the most horrific manner by the bite of his own dog!"

At Kilrush, County Clare, occurred a case of hydrophobia in March of 1844, which, from the misery and destitution into which were plunged the poor family in which it happened, excited at the time much and general interest. A boy, named Walsh, was bitten in February by a young dog which he had been rearing. No notice was taken of it till, in about four weeks after, the disease manifested itself in all intensity. What made the occurrence more melancholy was, that five others of the family, including the father, had been also bitten. The family were described as renting, at a pound per year, a quarter acre of land, on which they lived in a hovel, situated on the brink of a frightful sand-pit. Potatoes and salt, and occasionally a salted herring, had been their usual food. A horse, their sole means of support, which had been also bitten, died rabid. They could get no employment; they were deserted even by their relatives. Their neighbours were all afraid to approach them. The only person in communication with them was the dispensary physician of the district. These particulars, published in the newspapers, appeared to excite general commiseration. As at this time (1844) there was a marked increase of cases of hydrophobia in England, and in the north and south of Ireland, the daily press teemed with nostrums and prophylactics of all sorts, under the title of specifics; each was vouched for as infallible, with the same confidence as has been vouched for, of late years, the infallibility of each of certain specifics for Asiatic cholera; all who took it were saved,—all who did not take it, died. With others sent from different places to the family at Kilrush, one, which had been brought from Bohemia by Lord Clanwilliam, was sent from England, together with another specific—the best of all—the liberal donation of £20 by Lord Arundel, the present Duke of Norfolk.

In December 1846, according to the Limerick paper, "a fine boy, seven years old, son of Captain Kennedy, agent to the Devon estate, died of hydrophobia at Newcastle, near that city, from the bite of a stray cur-dog five weeks before."

In Castle Island, county of Kerry, in June 1844, "a poor woman died of hydrophobia, after three successive days of agonizing suffering, who had been bitten in the upper part of the wrist in May by a stray cur-dog, while driving some geese convenient to her house."

In Dungarvan, county of Waterford, "a poor woman, named Connor, died of hydrophobia in December 1844, from the bite of a small dog, which bit her several weeks before."

In the case of Garrett Nagle, who was bitten while at work on the repairs of a road, the bite, as mentioned above, was also inflicted by a small dog. The small dog, and the stray dog, may, in almost every instance, be taken to mean a cur-dog.* A peasant, returning home from a wedding party late at night, was bitten on the highroad by a stray dog, and died of hydrophobia. A boy, herding some sheep of his father's, was bitten in the hand by a stray dog, while in the act of defending the sheep from the dog, and died of hydrophobia. A poor girl, milking a cow in a field, the cow having been first bitten, was bitten by a stray dog, and died of hydrophobia. It is usual with dogs, in the commencement of rabies, to stray from home, biting frequently in their wanderings human beings and other animals. The loss to farmers and graziers in the destruction of "stock" by the bite of rabid dogs wandering through the country has been often grievous, if not ruinous. We may readily infer this from the accounts which occasionally appear in the public prints, of the ravages of a single mad dog among a flock or a herd. In the loss of his pig, or—as in the instance at Kilrush—of his horse, or as in other instances of his poultry, from the bite of a rabid dog, the poor cottier has, in Ireland, sometimes lost his all!

The following case may be interesting, as exemplifying the fact of the extreme shortness of interval (in this case only a very few days), which has sometimes elapsed between the infliction of the bite and the setting in of symptoms, contrasting widely with those instances recorded, in which the symptoms did not take place until twelve months or upwards after the bite. In the case of a peasant, which occurred several years since near Fermoy in this county, attended by the late Sir George Alley, physician to the Fermoy Dispensary, the symptoms, as I was informed by the resident medical officer of the Dispensary, did not manifest themselves until two years after

---

* Sir Walter Scott, in "Waverly", has given a graphic account of the nuisance of "innumerable cur-dogs" on the high roads of Scotland, at the time in which the scene of "Waverly" is laid.

the bite. In the present instance, a poor girl, washing clothes at "a slip" on the side of the river Bandon, was bitten by a stray hound belonging to a gentleman in the neighbourhood, which swam across the river from the opposite side, and landing at the spot where she was, bit her in the cheek. The disease, terminating fatally, came *on in three or four days after the bite.* She died in convulsions. The late Rev. T. R. England, who was my informant, hearing of her sickness, had some tea made for her ; but, upon its being offered to her to drink, he was surprised to hear that she refused it with marks of great aversion,—tea being regarded by the poorest class here, particularly in country places, as a luxury. Seeing a cut or scratch on her cheek, his suspicions were roused, which were the more awake from his having formerly seen a case of the disease, and from having read a good deal on the subject. Thus the fact of her having been bitten in the manner stated was elicited. She was, he said, an ignorant country girl, very young, who had probably never heard of such a disease. "Dread of water" is not a symptom in the rabid dog ; the supposition that it is has sometimes led to fatal errors. In the case of the peasant which occurred near Fermoy, the bite was inflicted in the thumb. The first symptom, on the disease manifesting itself, was numbness, and a pricking sensation as "from pins and needles" in and about the thumb. Died in forty-eight hours. Was so furiously delirious that it was necessary to "pinion him behind." The aversion to a smooth shining surface was very marked. A piece of looking-glass, which was stuck in the mud wall of the cabin—opposite to his bed place—had to be removed. The dog that bit him was wandering through the country, "raging mad," and bit dogs and pigs, that died rabid. "There is something surprising," says Van Swieten, "in the poison of this disease (hydrophobia), which will lie dormant for a long time without showing itself by any apparent signs, and yet, when it becomes active, raises a most acute disorder, which commonly kills before the fourth day."

